# ^13^C Metabolic Flux Analysis for Systematic Metabolic Engineering of *S. cerevisiae* for Overproduction of Fatty Acids

**DOI:** 10.3389/fbioe.2016.00076

**Published:** 2016-10-05

**Authors:** Amit Ghosh, David Ando, Jennifer Gin, Weerawat Runguphan, Charles Denby, George Wang, Edward E. K. Baidoo, Chris Shymansky, Jay D. Keasling, Héctor García Martín

**Affiliations:** ^1^Lawrence Berkeley National Laboratory, Biological Systems and Engineering Division, Berkeley, CA, USA; ^2^Joint BioEnergy Institute, Emeryville, CA, USA; ^3^Indian Institute of Technology (IIT), School of Energy Science and Engineering, Kharagpur, India; ^4^National Center for Genetic Engineering and Biotechnology (BIOTEC), Pathum Thani, Thailand; ^5^Department of Chemical and Biomolecular Engineering, University of California Berkeley, Berkeley, CA, USA; ^6^Department of Bioengineering, University of California Berkeley, Berkeley, CA, USA; ^7^Novo Nordisk Foundation Center for Biosustainability, Technical University Denmark, Horsholm, Denmark

**Keywords:** flux analysis, ^13^C metabolic flux analysis, -omics data, predictive biology, metabolic engineering

## Abstract

Efficient redirection of microbial metabolism into the abundant production of desired bioproducts remains non-trivial. Here, we used flux-based modeling approaches to improve yields of fatty acids in *Saccharomyces cerevisiae*. We combined ^13^C labeling data with comprehensive genome-scale models to shed light onto microbial metabolism and improve metabolic engineering efforts. We concentrated on studying the balance of acetyl-CoA, a precursor metabolite for the biosynthesis of fatty acids. A genome-wide acetyl-CoA balance study showed ATP citrate lyase from *Yarrowia lipolytica* as a robust source of cytoplasmic acetyl-CoA and malate synthase as a desirable target for downregulation in terms of acetyl-CoA consumption. These genetic modifications were applied to *S. cerevisiae* WRY2, a strain that is capable of producing 460 mg/L of free fatty acids. With the addition of ATP citrate lyase and downregulation of malate synthase, the engineered strain produced 26% more free fatty acids. Further increases in free fatty acid production of 33% were obtained by knocking out the cytoplasmic glycerol-3-phosphate dehydrogenase, which flux analysis had shown was competing for carbon flux upstream with the carbon flux through the acetyl-CoA production pathway in the cytoplasm. In total, the genetic interventions applied in this work increased fatty acid production by ~70%.

## 1 Introduction

In spite of several successes (Keasling and Chou, 2008; Goh et al., 2014), the production of renewable, economical, and environmentally sustainable fuels and chemicals from microbial fermentation remains challenging (Sims et al., 2010). There is a particular interest in the production of second-generation biofuels, which have the potential to provide significant environmental benefits in the form of reduced global dependence on crude oil and minimizing CO_2_ production (Naik et al., 2010). Second-generation biofuels and bioproducts also have higher energy densities and improved handling and performance characteristics (e.g., water miscibility) over ethanol produced from corn stocks (Fortman et al., 2008). Some types of second-generation biofuels can be produced from fatty acids produced through fermentation of sugars, in which the free fatty acids can be converted to alkanes by catalytic esterification or decarboxylation (Fjerbaek et al., 2009). Conversely, the host organism can be bioengineered to convert fatty acids into fatty acid ethyl esters [FAEE, Steen et al. (2010a)]. Moreover, unprocessed medium chain fatty acids (C6–C14) are commonly used in industrial applications as sources for bioproducts other than biofuels: lubricants, cosmetics, and pharmaceuticals. Free fatty acids can also be directly hydrogenated to form fatty alcohols (Voeste and Buchold, 1984).

Previous engineering attempts with *Saccharomyces cerevisiae* (Rodriguez et al., 2016) to produce fatty acid-derived biofuels (Runguphan and Keasling, 2014) from sugars have, for example, involved the overexpression of all three fatty acid biosynthesis genes, namely, acetyl-CoA carboxylase (*ACC1*), fatty acid synthase 1 (*FAS1*), and fatty acid synthase 2 (*FAS2*), as well as knocking out fatty acyl-CoA synthetases 1 and 4 (*FAA1* and *FAA4*). Altering the terminal converting enzyme in the engineered strain led to the production of free fatty acids at a titer of ~400 mg/L, fatty alcohols at ~100 mg/L, and fatty acid ethyl esters (biodiesel) at ~5 mg/L directly from simple sugars in shaking flask cultivation. More recent work (Zhou et al., 2016) reached a titer of 1 g/L of free fatty acids in shaking flask cultivation and 10.4 g/L in fed-batch cultivation. Besides blocking fatty acid activation and degradation and overexpressing *ACC1*, the cell was further engineered by introducing an optimized acetyl-CoA pathway and expressing a more efficient fatty acid synthase. In spite of this progress, higher yields, titer, and productivity are needed in order to obtain commercially viable strains. Furthermore, toward this goal it would be desirable to develop systematic methods. These systematic methods should not heavily rely on a detailed biochemical knowledge of the selected host or pathway but rather be generalizable and suggest non-intuitive engineering approaches.

Metabolic modeling provides a way to systematically determine genetic modifications that may improve yield. Flux-based metabolic modeling is particularly well suited for this endeavor since metabolic fluxes describe how carbon flows from feed to final product. Flux balance analysis (FBA) has previously been used successfully for this purpose (Asadollahi et al., 2009; Park et al., 2009). FBA obtains fluxes by using a network of cellular metabolism which includes all reactions, or at least as many as can be inferred from the genome through a metabolic reconstruction that yields a genome-scale stoichiometric model (Thiele and Palsson, 2010). This genome-scale model is coupled with a linear programing (LP) assumption that metabolism is tuned, due to evolutionary pressure, to maximize growth rate [or other evolutionary assumptions can be used, see Schuetz et al. (2007)]. Two-scale ^13^C metabolic flux analysis (2S-^13^C MFA) improves on FBA by retaining the genome-scale metabolic network but drops the evolutionary assumption in favor of ^13^C constraints from cellular metabolites measured experimentally (Martín et al., 2015). This is achieved by modeling fluxes at two different levels of resolution: for core metabolites and reactions, both stoichiometry and carbon labeling information are used, whereas for the remaining non-core metabolites and reactions, only stoichiometry is tracked, and their contribution to the core set labeling is considered to be negligible. This multiscale approach is valid as long as metabolic flux flows from core to peripheral metabolism and does not flow back, an assumption that is supported by the good fits between experimentally measured and computed labeling distributions obtained in general by ^13^C MFA methods thus far [which only consider core reactions, Moxley et al. (2009); Kajihata et al. (2015)]. 2S-^13^C MFA combines the informative constraints of ^13^C labeling experiments with genome-scale stoichiometry to improve the determination of metabolic fluxes and set confidence intervals based on experimental data. This method allows us to constrain metabolic fluxes without the need for carbon transitions for every reaction in the genome-scale model (Gopalakrishnan and Maranas, 2015a,b) and provides flux estimates for peripheral metabolism such as fatty acid production, which are the subject of this manuscript.

**Figure 1:**
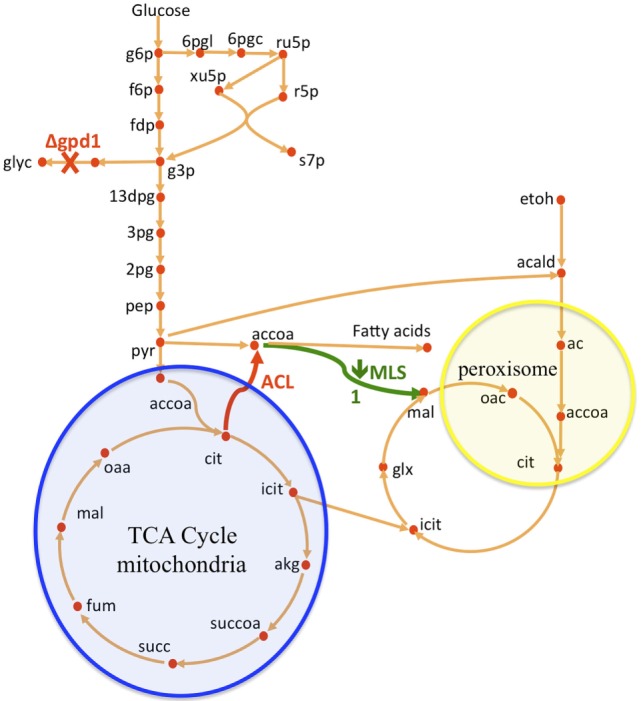
**Overview of *S. cerevisiae* metabolic pathways relevant to this study and metabolic interventions**. A non-native ATP citrate lyase (ACL) was introduced with the intention of increasing acetyl-CoA (accoa) supply, but fatty acid production did not increase significantly (Figure 2). The use of flux analysis suggested that more acetyl-coA was indeed produced, but that it was lost through malate synthesis (MLS, Figure 3). Downregulating this enzyme increased fatty acid (FA) production 26% (Figure 2). The use again of flux analysis suggested knocking out glycerol production (GPD1) for increased production. FA production improved an extra 33% (Figure 2). The mitochondrial and peroxisomal compartments are represented as blue and yellow circles, respectively. Metabolite abbreviations follow the BIGG database (Schellenberger et al., 2010).

In this paper, we improved a strain of *S. cerevisiae* (WRY2) that was previously constructed for free fatty acid production (Runguphan and Keasling, 2014). The previous metabolic interventions for this strain consisted of the overexpression of acetyl-CoA carboxylase and fatty acid synthases, and the elimination of FAA1 and FAA4 involved in the fatty acid degradation pathway of *S. cerevisiae*. Flux analysis guided further improvement of this strain (see Figure 1). First, we performed ^13^C tracer experiments on WRY2 so that we could apply 2S-^13^C MFA to determine fluxes in a genome-scale model of metabolism for our reference strain. We used this new approach to determine fluxes for WRY2 both before and after boosting acetyl-CoA production via the addition of ATP citrate lyase [ACL, Rodriguez et al. (2016)] from *Yarrowia lipolytica*. Although acetyl-CoA is the substrate for fatty acid production, the introduction of ACL resulted in only a small gain in fatty acid production of around 5%. 2S-^13^C MFA identified the most significant sink of acetyl-CoA after the introduction of ACL to be malate synthesis. After downregulating malate synthesis, we measured a significant increase in fatty acid production of 26%. Finally, as fatty acid production increased as we engineered WRY2 through both the addition of ACL and the downregulation of MLS, 2S-^13^C MFA showed that the glycerol-3-phosphate dehydrogenase (*GPD1*) pathway, which competes for carbon with the acetyl-CoA production pathway, was acting as a large carbon sink. We knocked out *GPD1* in our engineered strains so more carbon flux would be available for fatty acid production, and as expected, these strains had increased fatty acid production of 33%. In total, the genetic interventions applied in this work increased fatty acid production by ~70%.

## 2 Materials and Methods

### 2.1 Media, Cultivation, and Yeast Transformation

The parent strain for all genetic engineering, WRY2, was generated as described in Runguphan and Keasling (2014). For strain construction (see Table 1), precultures were grown with 5 mL of yeast extract + peptone + dextrose (YPD) medium in glass test tubes at 30°C with shaking at 200 rpm. After 18 h of growth, precultures were used to inoculate 50-mL cultures in 250-mL Erlenmeyer flasks. After 6 h of growth, strains were transformed by the lithium acetate method (Gietz and Woods, 2002) with linear DNA containing the genetic modification cassette as specified below. Transformed yeast cells were plated on non-selective YPD + 2% agar and were grown at 30°C for 18 h. The resulting cells were replica plated onto YPD agar plates + 200 mg/L of the following selective antibiotics: hygromycin B (X), nourseothricin (X), or G418 (X). Colonies were picked after 2 days and re-streaked on selective media. Integration of genetic modifications was confirmed by performing colony PCR on a single colony of the propagated strain.

**Table 1:** **List of strains and genotypes**.

Strain name	Genotype	Description	Reference	JBEI registry
BY4742	Mat*α*; his3Δ1; leu2Δ0; lys2Δ0; ura3Δ0	Direct descendent of S288C	Brachmann et al. (1998)	
WRY2	WRY2*	BY4742 where promoters of ACC1, FAS1 and FAS2 changed to TEF1 promoter and FAA1 and FAA4 were deleted		JBx_026085
WRY2 ACL	WRY2 ACL	WRY2 with ACL plasmid with TEF1 promoter	This study	JBx_048834
WRY2 ACL P*_TEF_*_1_-MLS1	WRY2 ACL; P*_TEF_*_1_*_m_*_2_-MLS1	WRY2 with ACL plasmid and downregulation of MLS1	This study	JBx_048836
WRY2 ΔGPD1	WRY2 gpd1Δ	WRY2 with the deletion of gpd1	This study	JBx_026430
WRY2 ΔGPD1 ACL	WRY2 gpd1Δ; ACL	WRY2 with ACL plasmid and the deletion of gpd1	This study	JBx_048838
WRY2 ΔGPD1 ACL P*_TEF_*_1_-MLS1	WRY2 gpd1Δ; ACL; P*_TEF_*_1_*_m_*_2_-MLS1	WRY2 with ACL plasmid plus deletion of gpd1 plus downregulation of MLS1	This study	JBx_048840

*Details are available in the public instance of the JBEI public registry (Ham et al., 2012), https://public-registry.jbei.org*.

WRY2* genotype: BY4742 FAA1Δ; FAA4Δ; acc1::P*_TEF_*_1_-ACC1; fas1::P*_TEF_*_1_-FAS1; fas2::P*_TEF_*_1_-FAS2.

### 2.2 Strain Construction

PCR amplification was performed using Prime STAR GXL DNA polymerase using the manufacturer’s instructions (Takara). Primers used in this study are listed in Table 2. Assemblies were performed using Gibson assembly master mix (New England Biolabs) and were transformed into DH10b competent cells for propagation. Plasmid DNA was purified using a QIAprep Spin Miniprep Kit (QIAGEN), and plasmids were sequenced with 2× coverage (Quintara). DNA sequences derived from *S. cerevisiae* were amplified from genomic DNA prepared using a modified Miniprep protocol: 1 mL of yeast cell culture in YPD medium was centrifuged in a screw cap tube (3000 × *g*) and resuspended in buffer P1 (from Qiagen kit). Cells were lysed by adding glass beads and shaking in a benchtop homogenizer/bead beating instrument (FastPrep-24, MP Biomedicals) for ~1 min. Resulting suspension was used for remaining steps in Qiagen Miniprep protocol.

**Table 2:** **List of primers used in this study**.

Modified gene	Template	F-primer	R-primer
GPD1 deletion	pAG32	TATATTGTACA	CATATAGGCATG
		CCCCCCC	AATATAT
		CCTCCACAAACA	TTTTATATATGTG
		CAAAT	TACACT
		ATTGATAATATAAA	GGGGCAAGGGAG
		Gttgcc	Aacggaa
		tcgtccccgccgg	agaagaaatggatcc
MLS1	pAG35-	ATTGTTTTGAACT	TTATCAACATCCA
downregulation	pTEF1m2	AAACA	CCAGT
		AAGTAGTAAAAGC	AATTTGACGTTAT
		ACATA	CCAAA
		AAAGAA TTAAGAAA	CTGACCTTAACC
		tcgac	ATtttttcta
		actggatggcggc	gaaaacttggatt

For construction of GPD1::hphMX4 (*GPD1* knockout), the hphMX4 cassette was amplified from pAG32 (Goldstein and McCusker, 1999) with primers containing 50 bp corresponding to the chromosomal sequence immediately 5′ and 3′ of the GPD1 locus.

For construction of NatMX3-PTEF1m2::PMLS1 (*MLS1* downregulation), the NatMX3-PTEF1m2 cassette was amplified from pAG35-PTEF1m2, a plasmid containing the NatMX3 cassette immediately 5′ of PTEF1m2. pAG35-PTEF1m2 was generated by restriction cloning as follows: a mutant variant of TEF1 promoter was amplified from p416-TEFm2 (Nevoigt et al., 2006) using primers flanked with restriction sites, and the resulting amplicon was ligated into the *Bam*HI and *Hin*dIII sites of pAG35 (Goldstein and McCusker, 1999).

For construction of ura3::P_PGK1_-*Yl*ACLb-P_TEF1_-*Yl*ACLa-kanMX4 (ACL addition), the P_PGK1_-*Yl*ACLb-P_TEF1_-*Yl*ACLa-kanMX4 cassette was generated by restriction digest of plasmid pCV278 with *Pme*I. pCV278 was generated by replacing the GAL1/10 promoters from pCV256 with PGK1/TEF1 promoters using a 3-fragment Gibson assembly reaction. The assembly was designed using Device Editor bioCAD software (Chen et al., 2012), and assembly primers were generated with j5 DNA assembly design automation software (Hillson et al., 2012) using the default settings. The fragment containing *Yl*ACLb-P_TEF1_-*Yl*ACLa-kanMX4 was amplified from pCD256, and the TEF1/PGK1 promoters were amplified from yeast genomic DNA.

Immediately preceding production experiments, all strains were transformed with pESC-Leu2d-’TesA and plated on -Leu. Colonies grew after 2 days and were used to inoculate minimal media with appropriate amino acid dropouts: CSM with 60 mg/L His, 90 mg/L Lys, 60 mg/L Ura, and 60 mg/L Met (HKUM media).

### 2.3 ^13^C Labeling Experiments

All liquid cultivations were carried out in minimal medium (1× yeast nitrogen base, 1.5% glucose, and 1M phosphate buffer in HKUM media). After precultivation overnight in glucose minimal medium, 30-mL cultures were inoculated to a starting OD_600_ of 0.05 and grown in 250-mL shake flasks at 30°C and 250 rpm. Aliquots were withdrawn during the exponential growth phase on glucose. For flux analysis experiments, natural abundance glucose was replaced by mixture of 80% of the 1-^13^C glucose and 20% of the U-^13^C isotopologue (^13^C-enrichment Z99%, Cambridge Usotope Laboratories, Andover). Sampling for metabolite labeling measurements for all the strains was done at 15 h: 6–8 mL of culture from liquid cultivations were removed for metabolites measurement using LCMS (see below). Extracellular metabolite concentrations were measured using HPLC at two time points: 13 and 15 h. These measurements were later converted into fluxes using OD values for different strains; the conversion factor from OD to cell dry weight (cdw) was 0.835.

### 2.4 Measurement of Labeling Patterns (Mass Distribution Vectors, MDVs)

For metabolite labeling samples, 5 mL of cell culture was pelleted at 8000 × *g* for 3 min at 4°C and resuspended in 300 μL of methanol, 300 μL of chloroform, and 250 μL of water. After vortexing, the suspension was transferred into 1.7-mL screw cap tube and 500 μL of beads were added. Bead beating was performed on samples for 10 s for 10 times with 1 min on ice between samples. Then, 350 μL of the aqueous layer was removed from the tube and filtered through a Millipore™ Amicon Ultra 3 kDa MW cut-off filter at 14,000 × *g* for 60 min at −2°C. Water was added to the flow-through to give a total volume of 1 mL. Following flash-freezing (with liquid nitrogen) then lyophilization, samples were reconstituted in 50 μL methanol–water (50:50, v/v) prior to analysis.

For measurement of intracellular amino acids, liquid chromatographic separation was conducted at 30°C with a Kinetex HILIC column (100-mm length, 4.6-mm internal diameter, 2.6-μm particle size; Phenomenex, Torrance, CA, USA) using a 1200 Series HPLC system (Agilent Technologies, Santa Clara, CA, USA). The injection volume for each measurement was 2 μL. The sample tray and column compartment were set to 6 and 40°C, respectively. The mobile phase was composed of 20 mM ammonium acetate in water (solvent A) and 10 mM ammonium acetate in 90% acetonitrile and 10% water (solvent B) (HPLC grade, Honeywell Burdick & Jackson, CA, USA). Ammonium acetate was prepared from a stock solution of 100-mM ammonium acetate and 0.7% formic acid (98–100% chemical purity, from Sigma-Aldrich, St. Louis, MO, USA) in water. Amino acids were separated with the following gradient: 90 to 70% B in 4 min, held at 70% B for 1.5 min, 70 to 40% B in 0.5 min, held at 40% B for 2.5 min, 40 to 90% B in 0.5 min, held at 90% B for 2 min. The flow rate was varied as follows: held at 0.6 mL/min for 6.5 min, linearly increased from 0.6 to 1 mL/min in 0.5 min, and held at 1 mL/min for 4 min. The total run time was 11 min. Mass spectrometry parameters can be found in Bokinsky et al. (2013).

Data acquisition and processing were performed by the MassHunter software package. The mass isotopomer distribution of the amino acid was obtained without fragmentation. From the mass isotopomer distribution of the amino acids, fluxes were calculated with the 2S-^13^C MFA software as described later. Labeling patterns were measured for the following intracellular amino acids: glycine (Gly), alanine (Ala), valine (Val), threonine (Thr), leucine (Leu), isoleucine (Ile), asparagine (Asp), glutamate (Glu), glutamine (Gln), arginine (Arg), phenylalanine (Phe), and tyrosine (Tyr).

### 2.5 Biomass and Extracellular Metabolite Concentrations

Biomass concentrations were determined by recording OD_600_ with a spectrophotometer (Novaspec II, Pharmacia Biotech, Uppsala, Sweden). Extracellular metabolite concentrations for ethanol, acetate, glycerol, and glucose were determined with an Agilent 1200 Series HPLC system equipped with a photodiode array detector set at 210, 254, and 280 nm (Agilent Technologies, Santa Clara, CA, USA). The separation of metabolites was conducted on an Aminex HPX-87H column with 8% cross linkage (150 mm length, 7.8 mm internal diameter, and 9 μm particle size; Bio-Rad, Richmond, CA, USA). A sample injection volume of 5 μL was used throughout. The sample tray and column compartment were set to 4 and 50°C, respectively. Isocratic elution was performed with 4-mM sulfuric acid at a flow rate of 0.6 mL/min. The HPLC system was equipped with a refractive index detector (Agilent Technologies), which was used to detect organic acids and glucose. Data acquisition and analysis were performed via Agilent Chemstation software. Biomass yields were obtained from a linear fit of substrate or byproduct concentrations during exponential growth as a function of corresponding biomass concentrations. Multiplication with the growth rate then yielded specific glucose uptake and byproduct secretion rates. The physiological parameters were determined from at least two independent biological replicates.

### 2.6 GC–MS Analysis of Free Fatty Acids

For free fatty acid production, strains were precultured in 5 mL aliquots in minimal medium (1× yeast nitrogen base, 1.5% glucose, and 1M phosphate buffer in HKUM media) over night and used to inoculate 30 mL minimal medium (1× yeast nitrogen base, 1.5% glucose, and 1M phosphate buffer in HLKUM media) in 250-mL flask cultures to achieve an initial OD_600_ of 0.05. After 96 h, 100 μL of yeast culture were spiked with 5 μL of pentadecanoic acid standard (3 mg/mL) and then mixed with 10 μL of 40% v/v tetrabutylammonium hydroxide (TBAH) solution (Sigma). Then, 100 μL of dichloromethane (DCM)/iodomethane (MeI) was added to the sample, and the mixture was agitated by vortex for 10 s. The organic (bottom) layer was transferred to a GCMS vial, and the solvent was allowed to evaporate completely. Then, 100 μL of fresh DCM was added to the extract, and the samples were run using a previously described method (Steen et al., 2010b) with some minor differences. The GC program was as follows: an initial temperature of 40°C was maintained for 3 min, followed by ramping to 250°C at a rate of 20°C/min, where the temperature was held for 5 min.

### 2.7 Flux Calculation

Fluxes were calculated through 2S-^13^C MFA (Martín et al., 2015), using code that is included as part of the Supplementary Material. Files containing all input information for 2S-^13^C MFA can be found therein: feed labeling information, measured extracellular fluxes, carbon transitions, measured labeling information, and SBML file for the genome-scale model [iMM904 (Mo et al., 2009)]. Detailed instructions on how to calculate fluxes and produce the figures in this manuscript can be found in the jupyter notebook provided as Supplementary Material.

For the purposes of fitting the measured labeling patterns, intracellular amino acids were assumed to be cytosolic, as has been assumed in previous studies (Moxley et al., 2009). Confidence intervals for fluxes were calculated through ^13^C flux variability analysis (^13^C FVA) by solving equations (16–23) in Martín et al. (2015). This procedure allowed us to calculate all fluxes compatible with the labeling data [equation (23) in Martín et al. (2015)], instead of only the fluxes that best fit the data, a piece of information of vital importance in order to produce valid conclusions. Confidence intervals are presented throughout the manuscript as, e.g., 0.5 [0.3–0.66], where 0.5 is the flux that best fits the available experimental data, 0.3 is the lowest flux that is compatible with the data, and 0.66 is the highest flux compatible with the data. External labeling variability analysis (ELVA) was performed and used to decide the size of the core set of reactions, as explained in Martín et al. (2015).

## 3 Results

### 3.1 ACL Alone Improves Fatty Acid Production Minimally

ATP citrate lyase (ACL) is an enzyme, which is not normally present in *S. cerevisae* (Rodriguez et al., 2016), but which in other organisms such as plants or oleaginous yeast produces additional cytosolic acetyl-CoA, which further acts as a precursor in the production of fatty acids or many thousands of other specialized metabolites including waxes, sterols, and polyketides. In the presence of ATP and coenzyme A in the cytoplasm, ACL catalyzes the cleavage of citrate to yield acetyl-CoA, oxaloacetate, ADP, and orthophosphate (see Figure 1):

$$\text{citrate}+\text{ATP}+\text{CoA}+{\text{H}}_{\text{2}}\text{O}\to \text{oxaloacetate}+\text{acetyl}–\text{CoA}+\text{ADP}+\text{Pi}$$

**Figure 2:**
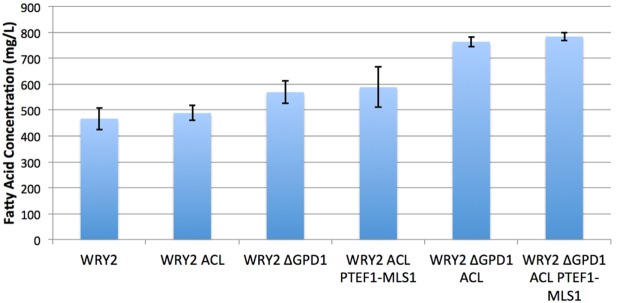
**Fatty acid production for the various strains studied in this manuscript**. *S. cerevisiae* WRY2 is the base strain used for these studies. The addition of ACL was expected to increase acetyl-CoA availability but did not increase final production. However, the downregulation of MLS did increase production, as suggested by flux analysis. The highest production was obtained by knocking out glycerol production, improving production in the overall engineering process by 70%. Fatty acid measurements shown here were performed at the end of 100 h, and the error bars represent the SD obtained for three replicates.

As the ACL enzyme produces acetyl-CoA precursors (Rodriguez et al., 2016), we introduced *ACLY* (from *Y. lipolytica*) containing plasmids to our WRY2 strain (see Methods) to increase the production of acetyl-CoA in WRY2, as has been shown to be the case for the production of *n*-butanol (Lian et al., 2014). This resulted in a small 5% increase in fatty acid production (Figure 2). In order to investigate why the expected increase in acetyl-CoA production had not resulted in higher fatty acid production, we used 2S-^13^C MFA.

### 3.2 2S-^13^C MFA Indicates Acetyl-CoA Is Diverted from Fatty Acid Metabolism via Malate Synthase

**Figure 3:**
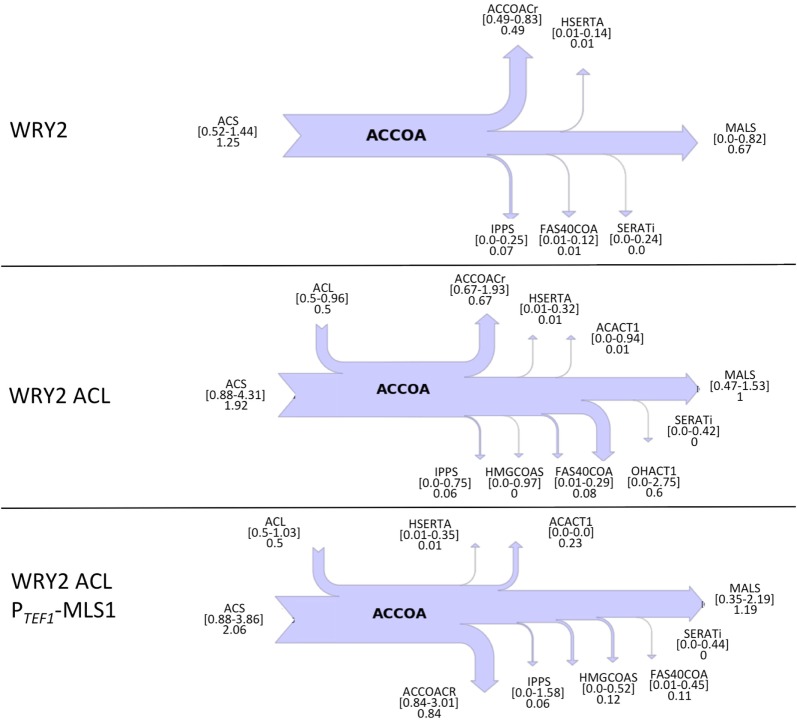
**Cytosolic acetyl-CoA balances obtained from 2S-^13^C MFA for WRY2, WRY2 ACL, and WRY2 ACL P_TEF1_-MLS1**. Flux of acetyl-CoA (accoa) producing and consuming reactions is shown as a sankey diagram. Reactions on the left of the diagram produce acetyl-coA and reactions on the right consume it, with arrow size indicating total flux. Reaction and metabolite names follow the BIGG database conventions (Schellenberger et al., 2010). Numbers below the reaction names indicate best fits to data and confidence intervals. For example, the acetyl-coA synthetase (ACS) for WRY2 shows that the flux that best fits the data is 1.25, but the flux could be any value between 0.52 and 1.44 (confidence intervals). The top diagram (WRY2) shows that all acetyl-coA is produced by ACS and MALS may act as a sink, although this is not assured (lower confidence interval is 0). Once the ACL is added (WRY2 ACL), total acetyl-coA increases, but the carbon sink into MALS becomes certain (lower confidence interval 0.47 >0), which is consistent with the lack of fatty acid production increase (Figure 2). Downregulation of MALS (WRY2 ACL P_TEF1_-MLS1) seems to have produced an increase in flux toward fatty acid metabolism, for which the reaction ACCOACr is the first step. The confidence intervals for ACCOACr before and after the downregulation ([0.67–1.93] vs. [0.84–3.01]) are too wide to make this conclusion, but the increase in fatty acid production (Figure 2) indicates this to be the case.

To diagnose and remedy the small increase in fatty acid production in the face of acetyl-CoA substrate production increases via the addition of ACL-containing plasmids to our WRY2 strain, we performed 2S-^13^C MFA to determine acetyl-CoA substrate fates. Fluxes for reactions producing and consuming acetyl-CoA for the engineered fatty acid producing strain WRY2 and the WRY2 strain with ACL can be found in Figure 3, showing a genome-wide balance as determined by 2S-^13^C MFA. The total amount of acetyl-CoA produced by strain WRY2 ACL (2.42 mmol/gdw/h) seems to almost double that of strain WRY2 (1.25 mmol/gdw/h) due to acetyl-CoA production addition by ACL of 0.5 mmol/gdw/h and an increase in acetyl-CoA production by acetyl-CoA synthetase (ACS) of ~0.7 mmol/gdw/h. However, these flux estimates have very large confidence intervals: 0.52–1.44 mmol/gdw/h for ACS flux where the best fit is 1.25 mmol/gdw/h for the WRY2 strain, and 1.38–4.85 mmol/gdw/h for the addition of ACS and ACL flux where 2.42 is the addition of best fits for the WRY2 strain with ACL. The confidence intervals represent maximum and minimum values of this flux compatible with the ^13^C labeling data (see Methods). Hence, the real flux for ACS in the WRY2 strain could be anywhere between 0.52 and 1.44, but the best fit for the data is 1.25, and similarly, the real flux for the addition of ACS and ACL could be anywhere in the interval 1.38–4.85 but our best guess is 2.42, based on the data. These wide confidence intervals represent the fact that, for a genome-scale model, metabolites can follow a variety of pathways to a given destination, and the available experimental data (metabolite labeling and measured exchange fluxes) cannot determine fully which ones are being used. This multiplicity of available pathways can be captured by 2S-^13^C MFA but not by ^13^C MFA (Martín et al., 2015). In this way, total acetyl-coA flux has changed from a value between 0.52 and 1.44 to a value between 1.38 and 4.85 (which may mean no change) where our best fits indicate a doubling from 1.25 to 2.42. However, we will see that in spite of these large confidence intervals, we can still use this information to guide metabolic engineering efforts to increase production (Figure 2). Hence, the addition of ACL seems to have increased total acetyl-CoA substrate production, but the data suggest that this effect is offset by an increase in malate synthase (MALS) consumption of acetyl-CoA of around 1.0 mmol/gdw/h ([0.47–1.53] conf. interval), with not much flux rerouted to acetyl-CoA carboxylase (ACCOACr, gateway to fatty acid metabolism): 0.49 mmol/gdw/h ([0.49–0.83] conf. interv.) vs. 0.67 mmol/gdw/h ([0.67–1.93] conf. interv.). This is consistent with the small increase in fatty acid production after adding ACL (Figure 2).

Since flux analysis indicated that the extra acetyl-CoA provided by the ACL was being shuttled into the MALS reaction, we decided that our next engineering step would be to downregulate the gene corresponding to MALS in the hope that this would increase the carbon flux toward fatty acid synthesis. Although the gene *MLS1* which encodes cytosolic malate synthase has been shown to be transcribed mostly during growth on C_2_ carbon sources, *MLS1* transcription has also been observed during growth on glucose (Regenberg et al., 2006). Therefore, deletion or downregulation of *MLS1* should contribute to increasing the cytosolic acetyl-CoA supply. We found that knocking out *MLS1* resulted in a very slowly growing strain (perhaps due to acetyl-CoA accumulation, which may be toxic due to protein acetylation). This effect was not of interest to us since we are focused on total fatty acid production, unlike Krivoruchko et al. (2013), who found a *MLS1* knockout to be key part of an engineering strategy to increase acetyl-CoA-based production of butanol titers by 6.5-fold. However, we found that downregulating *MLS1* (Methods), instead of knocking it out, resulted in viable strains and an immediate fatty acid production increase of ~26% (Figure 2). While this is consistent with our previous deduction that the MALS reaction is a significant carbon sink, the confidence intervals for MALS flux before and after the downregulation ([0.47–1.53] vs. [0.35–2.19], Figure 3) are too wide to confirm that MALS flux has indeed decreased. Although the best fit values (1.0 vs. 1.19) have increased, the confidence intervals provide the range of all possible fluxes compatible with available experimental data and are not narrow enough to confirm or discard that MALS flux has decreased. This case highlights the strength of our analysis, which allows us to judge the extent of validity of our inferences and when it is appropriate to derive further conclusions.

### 3.3 GPD1 Knockout Improves Fatty Acid Production

Glycerol-3-phosphate dehydrogenase (encoded by *GPD1*) catalyzes the conversion of dihydroxyacetone phosphate to glycerol 3-phosphate and plays an important role in the synthesis of lipids. Furthermore, it competes for carbon flux with the acetyl-CoA-based fatty acid synthesis pathways whose production levels we are attempting to maximize (Figure 1). Using 2S-^13^C MFA, we determined that flux through reaction GPD1ir [reaction abbreviations follow the BIGG database (Schellenberger et al., 2010)] in the WRY2, WRY2 ACL, and WRY2 ACL P_TEF1_-MLS1 strains to be 2.45 [2.45–2.45], 0.6 [0.68–0.685], and 1.68 [1.2–1.69] mmol/gdw/h. Therefore, as we engineered WRY2 for greater free fatty acid production flux, the competing glycerol-3-phosphate dehydrogenase pathway deviated carbon away from fatty acid production. If this competing carbon flux could be decreased by knocking out *GPD1* more carbon flux might be available for fatty acid production. As expected, WRY2 ΔGPD1 and WRY2 ΔGPD1 ACL strains had higher fatty acid production over WRY2 and WRY2 ACL of 22 and 56%, respectively (Figure 2).

**Figure 4:**
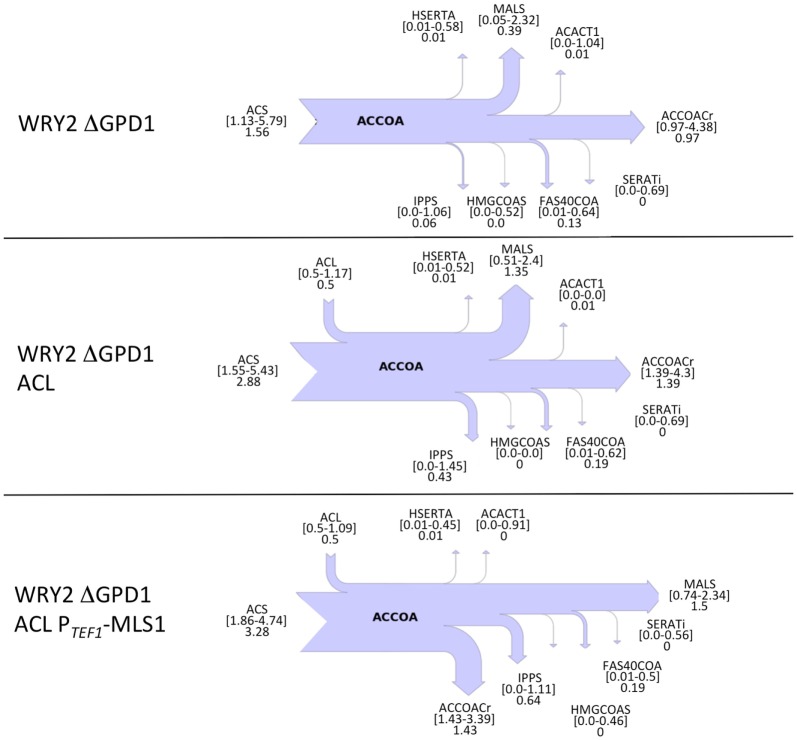
**Cytosolic acetyl-CoA balances obtained from 2S-^13^C MFA for strains with *GPD1* knocked out**. Flux of acetyl-CoA (accoa) producing and consuming reactions is shown as a sankey diagram as in Figure 3. In spite of the large confidence intervals for the calculated fluxes, several conclusions can be drawn. The total flux through ACCOACr, for example, is doubled through the full engineering process presented here: compare [0.49–0.83] for WRY2 in Figure 3 versus [1.43–3.39] for WRY2 ΔGPD1 ACL P_TEF1_-MLS1 in this figure. Also, the effect of the GPD1 knockout definitely increases flux through ACCOACr ([0.49–0.83] for WRY2 in Figure 3 versus [0.97–4.38] for WRY2 ΔGPD1). Furthermore, the effect of ACL involves the definite activation of reaction MALS as a carbon sink (similar effect as in Figure 3).

**Figure 5:**
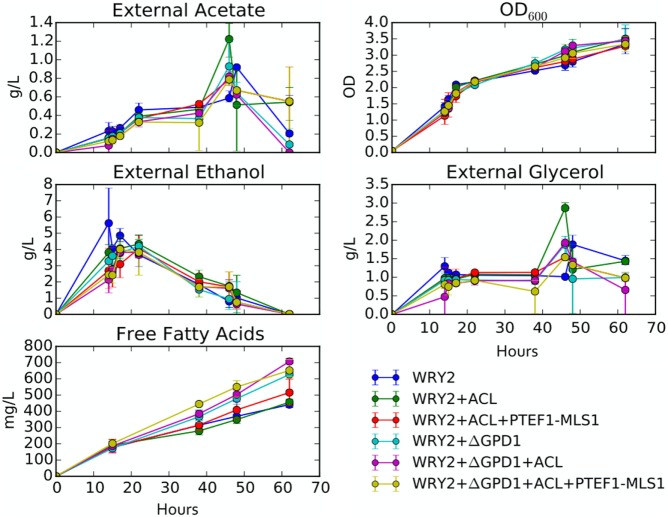
**Growth rates and external metabolite concentrations: shown are the external metabolite concentrations for ethanol, glycerol, and acetate for the strains studied in this manuscript**. Biomass for these strains is displayed in terms of optical density at 600 nm (OD_600_). All measurements are an average over three biological replicates.

Confirming our intuitions that knocking out *GPD1* allows for more carbon flux into acetyl-CoA-based fatty acid synthesis, 2S-^13^C MFA flux profiles on our *GPD1* knockout strains suggest increased acetyl-CoA production (Figure 4). In spite of wide confidence intervals for fluxes, we can see that the total flux into fatty acid production (ACCOACr in Figures 3 and 4) is doubled through the engineering process presented here ([0.49–0.83] for WRY2 in Figure 3 versus [1.43–3.39] for WRY2 ΔGPD1 ACL P_TEF1_-MLS1 in Figure 4). As expected, less glycerol is produced by the strains where *GPD1* was knocked out (Figure 5).

### 3.4 Growth Rates and External Metabolite Concentrations

As we have engineered WRY2 for greater fatty acid production, we found that growth rates and final biomass after 62 h did not change very significantly (Figure 5). In total, we were able to bring fatty acid production from 460 to 780 mg/L, a 70% increase, through the addition of ACL, downregulation of MALS, and knockout of *GPD1*, yet this metabolic burden was not large enough to slow down the growth rate of our strains. Similarly, external metabolite concentrations between strains were qualitatively similar, with measured metabolite concentrations averaged over three biological replicates shown over time in Figure 5.

## 4 Discussion

In this study, we have shown that we can use flux profiles obtained from 2S-^13^C MFA to guide and troubleshoot a bioengineering process aimed at increasing product yield. The flux profiles obtained from 2S-^13^C MFA describing acetyl-CoA balances (believed to be the limiting factor) have provided actionable insights for metabolic engineering efforts that have culminated in a 70% increase in fatty acid production. While the confidence intervals in the fluxes are large, they can still be successfully used to guide engineering efforts. Initially, the base strain was complemented with ACL in the hope of producing more acetyl-CoA. The flux profiles suggest that ACL is effective in increasing acetyl-CoA production, in the order of ~0.5 mmol/gdw/h higher than that for the WRY2 strain alone. However, this extra acetyl-CoA supply is not routed into fatty acid production but, rather, diverted into malate production through MALS. By downregulating the activity of malate synthase, we were able to increase fatty acid production by 26%. Similarly, carbon loss through GPD1 suggested that knocking out this reaction would increase production. This knockout resulted in increased acetyl-CoA creation, as well as a fatty acids production increase of 70% over the reference strain, when combined with ACL and MALS downregulation.

Free fatty acid production levels can probably be increased further. Metabolite and biomass measurements are qualitatively very similar across our engineered strains, indicating that the metabolic burden of producing free fatty acids at the production levels achieved in this manuscript are not severe even as we increased fatty acid production by 70%. Growth rates have not yet been affected by the metabolic engineering steps taken so far, which likely indicates that maximum production levels have not been reached.

A possible way to increase production further involves using a different type of ACL gene. The origin of the ACL gene used in the manuscript is *Y. lipolytica*, which is an obligate aerobic, oleaginous yeast capable of accumulating large amounts of lipids, predominately of the triacylglycerol type (Papanikolaou et al., 2002). Although ACL from *Y. lipolytica* has previously been shown to provide ATP citrate lyase activity when expressed in *S. cerevisiae* plasmids (Rodriguez et al., 2016), ACL from *Aspergillus nidulans* is known to be roughly five times more active in the cytoplasm (Rodriguez et al., 2016) of *S. cerevisase*. Significant increases in fatty acid production might be possible if the strains studied in this manuscript are recreated with the ACL gene from *A. nidulans*, as has been shown to be the case when using a ACL from *Mus musculus* (Zhou et al., 2016). However, it must be mentioned that this production increase was obtained when the *M. musculus* ACL was combined with further engineering in acetyl-CoA supply and a more efficient fatty acid synthase.

Another strategy to improve production might involve a pyruvate dehydrogenase (PDH) bypass (Kozak et al., 2014; Lian et al., 2014). Our results are consistent with the availability of the fatty acid precursor acetyl-CoA as a limiting factor to fatty acid production for the *S. cerevisiae* strains studied in this manuscript. Acetyl-CoA metabolism is strongly compartmentalized in yeast, separated into four spatial regions, the cytosol, mitochondria, peroxisomes, and nucleus. Acetyl-CoA in the cytoplasm is produced via the substrate acetaldehyde, formed by the decarboxylation of pyruvate. Unfortunately, a large part of the glycolytic flux is directed toward ethanol production due to the Crabtree effect (Van et al., 1998) when grown on glucose. This limits the availability of acetyl-CoA in the cytosol, with earlier research showing that strategies such as engineering the PDH bypass in *S. cerevisiae* enhanced the cytosolic acetyl-CoA supply, resulting in increased production of acetyl-CoA-derived products such as the isoprenoids amorphadiene (Shiba et al., 2007) and *α*-santalene (Chen et al., 2013), and polyhydroxybutyrate (Kocharin et al., 2012). A similar engineering strategy could be adopted to further improve fatty acid production in our highest yielding strains.

## Author Contributions

AG, WR, EB, GW, CD, and JG performed experiments. AG did the flux calculations. DA, HM, AG, JK, and CD wrote the paper.

## Conflict of Interest Statement

JK has financial interests in Amyris and Lygos. The remaining authors declare that the research was conducted in the absence of any commercial or financial relationships that could be construed as a potential conflict of interest.

## Supplementary Material

Additional File 1 – Zip Files Including Code Files and 2S-^13^C MFA Jupyter Notebook

Code used to produce the results (requires GAMS and CONOPT licenses), along with a jupyter notebook [Perez and Granger (2007), www.jupyter.org] that produces all the flux figures used in this manuscript.
