# Supplementary material for: 13C Metabolic Flux Analysis for Systematic Metabolic Engineering of S. cerevisiae for Overproduction of Fatty Acids
Source: Front Bioeng Biotechnol. 2016 Oct 5;4:76. doi: 10.3389/fbioe.2016.00076 (PMC5050205; doi:10.3389/fbioe.2016.00076)
Supplement: Supplementary file 1 [file Data_Sheet_1.ZIP › Ghosh2016Dir/Ghosh et al 2016 notebook.html]

Ghosh et al 2016 notebook


# Jupyter Notebook for "$^{13}$C Metabolic Flux Analysis for systematic metabolic engineering of *S. cerevisiae* for overproduction of fatty acids" Ghosh *et al* 2016¶

# Setup¶

Loading necessary modules and setting up needed paths:

This is the only path that needs to be changed in this whole notebook, set it to whichever directory you decompressed the supplementary material (preferably a directory without NFS mounting, to avoid delays):

In [1]:

```
pythonPath = "/scratch/hgmartin_scratch/Ghosh2016Dir"
```

In [2]:

```
import os,sys
os.chdir(pythonPath)

if pythonPath not in sys.path:
    sys.path.append(pythonPath)
os.environ["QUANTMODELPATH"] = pythonPath
%matplotlib inline
```

In [3]:

```
import SBMLclasses, FluxModels

dirDATA           = pythonPath+'/data/'
```

Test that GAMS and CONOPT are installed:

In [4]:

```
status = os.system('gams')
if status == 0:
    print "GAMS call working."
else:
    print "GAMS is not installed."
```

```
GAMS call working.
```

# Auxiliary functions¶

This auxiliary function assembles the final 2S $^{13}$C MFA SBML file from the genome-scale model SBML file, the transitions, the feed information, the measured labeling data and the measured extracellular fluxes:

In [5]:

```
def getSBMLfile(strain):
    ACLstrains = ['awry1','amgpd1','agpd1','amwry1']
    inputType = 'Glc'

    
    REACTIONSfilename = dirDATA + strain + '/REACTIONS'+strain+inputType+'.txt' 
    FEEDfilename      = dirDATA + strain + '/FEED'+strain+inputType+'.txt'
    CEMSfilename      = dirDATA + strain + '/LCMS'+strain+inputType+'.txt'    
    FLUXESfilename    = dirDATA + strain + '/FLUX'+strain+inputType+'.txt'
    if strain in ACLstrains:
        SBMLfilename      = pythonPath+'/iMM904TKACL.xml'
    else:
        SBMLfilename      = pythonPath+'/iMM904TKs.xml'
    
    
    reacNet = SBMLclasses.TSReactionNetwork(SBMLfilename)
    reacNet.changeModelName('SciMM904'+strain)        
        
    reacNet.loadFluxBounds(FLUXESfilename)
        
    reacNet.addReactions(REACTIONSfilename,translate2SBML=True)        
        
    reacNet.addLabeling(CEMSfilename,'LCMS',minSTD=0.001)        
    
    reacNet.addFeed(FEEDfilename)    

    TSSBMLfile = ('SciMM904'+strain+inputType+'TS.xml',reacNet.write('toString'))
    
    return TSSBMLfile
```

This function obtains the names of fluxes for which we will be calculating flux confidence intervals:

In [6]:

```
def getFluxNames(TSmodel):
    coreFluxes = TSmodel.ReacNet.C13ReacNet.reactionList.getReactionNameList(level=1)
    fluxNames = [name for name in coreFluxes if not 'EX' in name]
    fluxNames.extend(['G3PD1ir','OHACT1','ACACT1','HMGCOAS'])
    fluxNames = list(set(fluxNames)) # Eliminate redundancies
    
    return fluxNames
```

# WRY2¶

Obtain fluxes for WRY2:

In [7]:

```
TSSBMLfile = getSBMLfile('wry1')
TSmodel  = FluxModels.TwoSC13Model(TSSBMLfile)
TSresult = TSmodel.findFluxesRanges(Nrep=30,fluxNames=getFluxNames(TSmodel))
```

Plot the fits:

In [8]:

```
TSresult.plotExpvsCompLabelFragment(titleFig='wyr2Glc')
```

Plot the ELVA:

In [9]:

```
TSresult2  = TSmodel.ELVA(TSresult, erase=False)  
TSresult2.plotExpvsCompLabelxvsy(titleFig='Glc',outputFileName="ELVAComparisonGlc.txt")
```

Sankey plot for aceytul-CoA:

In [10]:

```
TSresult.plotMetaboliteFlux('accoa_c',titleFig='WRY2',minFluxGroup = 0.001, maxFluxGroup=1.6)
```

Flux flowing through Glycerol-3-phosphate dehydrogenase:

In [11]:

```
fluxDict = TSresult.ReacNet.reactionList.getFluxDictionary(rangeComp='all')
print fluxDict['G3PD1ir']
```

```
Forward: NA
Backward: NA
Net: [2.45 : 2.455928 : 2.45593]
Exchange: NA
```

# WRY2 + ACL¶

In [12]:

```
TSSBMLfile = getSBMLfile('awry1')
TSmodel  = FluxModels.TwoSC13Model(TSSBMLfile)
TSresult = TSmodel.findFluxesRanges(Nrep=30,fluxNames=getFluxNames(TSmodel))
```

In [13]:

```
TSresult.plotExpvsCompLabelFragment(titleFig='awyr1')
```

In [14]:

```
TSresult2  = TSmodel.ELVA(TSresult, erase=False)  
TSresult2.plotExpvsCompLabelxvsy(titleFig='Glc',outputFileName="ELVAComparisonGlc.txt")
```

In [15]:

```
TSresult.plotMetaboliteFlux('accoa_c',titleFig='aWRY1',minFluxGroup = 0.001, maxFluxGroup=1.6)
```

In [16]:

```
fluxDict = TSresult.ReacNet.reactionList.getFluxDictionary(rangeComp='all')
print fluxDict['G3PD1ir']
```

```
Forward: NA
Backward: NA
Net: [0.68 : 0.684693 : 0.68494]
Exchange: NA
```

# WRY2 + dGPD1¶

In [17]:

```
TSSBMLfile = getSBMLfile('gpd1')
TSmodel  = FluxModels.TwoSC13Model(TSSBMLfile)
TSresult = TSmodel.findFluxesRanges(Nrep=30,fluxNames=getFluxNames(TSmodel))
```

In [18]:

```
TSresult.plotExpvsCompLabelFragment(titleFig='gpd1')
```

In [19]:

```
TSresult2  = TSmodel.ELVA(TSresult, erase=False)  
TSresult2.plotExpvsCompLabelxvsy(titleFig='Glc',outputFileName="ELVAComparisonGlc.txt")
```

In [20]:

```
TSresult.plotMetaboliteFlux('accoa_c',titleFig='gpd1',minFluxGroup = 0.001, maxFluxGroup=1.6)
```

# WRY2 + ACL + down MLS1¶

In [21]:

```
TSSBMLfile = getSBMLfile('amwry1')
TSmodel  = FluxModels.TwoSC13Model(TSSBMLfile)
TSresult = TSmodel.findFluxesRanges(Nrep=30,fluxNames=getFluxNames(TSmodel))
```

In [22]:

```
TSresult.plotExpvsCompLabelFragment(titleFig='amwry1')
```

In [23]:

```
TSresult2  = TSmodel.ELVA(TSresult, erase=False)  
TSresult2.plotExpvsCompLabelxvsy(titleFig='Glc',outputFileName="ELVAComparisonGlc.txt")
```

In [24]:

```
TSresult.plotMetaboliteFlux('accoa_c',titleFig='amwry1',minFluxGroup = 0.001, maxFluxGroup=1.6)
```

In [25]:

```
fluxDict = TSresult.ReacNet.reactionList.getFluxDictionary(rangeComp='all')
print fluxDict['G3PD1ir']
```

```
Forward: NA
Backward: NA
Net: [1.2 : 1.685187 : 1.68519]
Exchange: NA
```

# WRY2 + dGPD1 + ACL¶

In [26]:

```
TSSBMLfile = getSBMLfile('agpd1')
TSmodel  = FluxModels.TwoSC13Model(TSSBMLfile)
TSresult = TSmodel.findFluxesRanges(Nrep=30,fluxNames=getFluxNames(TSmodel))
```

In [27]:

```
TSresult.plotExpvsCompLabelFragment(titleFig='agpd1')
```

In [28]:

```
TSresult2  = TSmodel.ELVA(TSresult, erase=False)  
TSresult2.plotExpvsCompLabelxvsy(titleFig='Glc',outputFileName="ELVAComparisonGlc.txt")
```

In [29]:

```
TSresult.plotMetaboliteFlux('accoa_c',titleFig='agpd1',minFluxGroup = 0.001, maxFluxGroup=1.6)
```

# WRY2 + ACL + dGPD1+ down MLS1¶

In [30]:

```
TSSBMLfile = getSBMLfile('amgpd1')
TSmodel  = FluxModels.TwoSC13Model(TSSBMLfile)
TSresult = TSmodel.findFluxesRanges(Nrep=30,fluxNames=getFluxNames(TSmodel))
```

In [31]:

```
TSresult.plotExpvsCompLabelFragment(titleFig='amgpd1')
```

In [32]:

```
TSresult2  = TSmodel.ELVA(TSresult, erase=False)  
TSresult2.plotExpvsCompLabelxvsy(titleFig='Glc',outputFileName="ELVAComparisonGlc.txt")
```

In [33]:

```
TSresult.plotMetaboliteFlux('accoa_c',titleFig='amgpd1',minFluxGroup = 0.001, maxFluxGroup=1.6)
```
